# Select updates from ASCO and ESMO 2024 for gastrointestinal cancer care

**DOI:** 10.1093/oncolo/oyaf020

**Published:** 2025-02-25

**Authors:** Tim Jang, George P Kim, Thomas J George

**Affiliations:** University of Florida Health Cancer Center, Gainesville, FL 32608, United States; Department of Medicine, University of Florida College of Medicine, Gainesville, FL 32608, United States; University of Florida Health Cancer Center, Gainesville, FL 32608, United States; Department of Medicine, University of Florida College of Medicine, Gainesville, FL 32608, United States; University of Florida Health Cancer Center, Gainesville, FL 32608, United States; Department of Medicine, University of Florida College of Medicine, Gainesville, FL 32608, United States

**Keywords:** ASCO Annual Meeting, gastrointestinal malignancy, ESMO Congress, colorectal cancer, esophagogastric cancer, circulating tumor DNA (ctDNA), clinical trials

## Abstract

**Background:**

Gastrointestinal (GI) malignancies remain the culprit behind a substantial portion of cancer-related mortality worldwide, and outcomes for patients with advanced or metastatic GI cancers remain poor despite continued efforts to improve care. In 2024, the ongoing clinical trials to optimize and improve GI cancer care showcased progress in molecular diagnostics, systemic therapies, and localized treatment approaches, providing hope for continued progress toward improved patient outcomes.

**Materials and Methods:**

This review summarizes selected updates in GI cancer care from the 2024 ASCO and ESMO Annual Meetings, including both positive and negative trials that, while not universally practice-changing, contribute to shaping GI cancer care, clinical management, or address key questions in the field. The selected trials cover early detection and diagnostic advances, perioperative management, metastatic disease management, and immune checkpoint inhibitor (ICI)’s emerging role in GI cancer.

**Results:**

Various clinical trials in perioperative management and their results continue to reshape or strengthen the current treatment paradigms. The use of ICIs for microsatellite instability-high colorectal cancer (CRC) presented a notable advancement with the potential to improve patient outcomes. Localized treatments such as thermal ablation appear to benefit some patients with CRC and liver metastases.

**Conclusions:**

The collection of trial results presented at the 2024 ASCO and ESMO Annual Meetings denote the ongoing efforts of the medical and scientific community for optimizing GI cancer care. The ongoing efforts of the GI cancer research and patient community provide hope for continued progress toward improved patient outcomes and new standards of care.

**Implications for Practice**

This review summarizes key updates from selected phase II/III trials presented at the 2024 ASCO and ESMO Annual Meetings. This review includes both positive and negative trials that, while not universally practice-changing, contribute to shaping gastrointestinal (GI) cancer care, clinical management, or address key questions in the field. We believe that this review will serve as a clinical resource covering some of the latest GI cancer trial results and their potential implications in current GI cancer care.

## Introduction

Gastrointestinal (GI) malignancies represent nearly a dozen different cancers and various tumor histologies originating within the digestive tract system. They remain one of the prime causes of cancer-related mortality worldwide.^1^ In 2024, it was estimated that there would be over 350 000 new diagnoses of GI cancers in the United States alone, with more than 170 000 deaths from GI malignancies.^2^ The dismal reality is that prognosis remains guarded for patients with advanced or metastatic disease given their poor overall survival.^3,4^ The collaborative efforts of investigators conducting clinical trials have sought ways to enhance early detection, improve localized treatments, and develop more effective systemic therapies. This review provides a concise summary of ongoing investigations in the field that were presented at the 2024 ASCO and ESMO Annual Meetings. In selecting the trials for this review, we prioritized primarily phase II/III studies presented or published at ASCO or ESMO 2024 that were considered clinically relevant due to their scale, design, and/or implications for future research (Table 1). This includes both positive and negative trials that, while not universally practice-changing, contribute to shaping GI cancer care, clinical management, or address key questions in the field. While a complete review of all clinically relevant trials is beyond the scope of this review, and the level of data presented requires more thorough analysis through peer-reviewed publication, we believe that this review will help update clinicians on the latest GI cancer trial results and their potential implications in current GI cancer care.

**Table 1.** Selected recent studies in GI cancer presented at ASCO and/or ESMO 2024.

Study name	Type of study	Results
ECOG-ACRIN EA2174	Randomized Phase II/III Clinical Trial	In locoregional E/GEJ adenocarcinoma, the addition of neoadjuvant nivolumab to standard chemoradiation did not improve the pCR rate.
COLLISON	Randomized Phase III Clinical Trial	For colorectal liver metastases ≤ 3cm, thermal ablation was noninferior compared with surgical resection in terms of local control and overall survival.
ESOPEC	Randomized Phase III Clinical Trial	In patients with resectable locally advanced esophageal adenocarcinoma, perioperative FLOT and surgery improved survival compared with a neoadjuvant chemoradiation approach.
ORCHESTRA	Randomized Phase III Clinical Trial	Additional tumor debulking following standard first-line palliative systemic therapy does not improve OS in patients with multiorgan metastatic colorectal cancer.
ARMANI	Randomized Phase III Clinical Trial	In patients with HER2-negative metastatic G/GEJ cancer not eligible for immune checkpoint inhibitor therapy, switch maintenance with ramucirumab plus paclitaxel after 3 months of oxaliplatin-based doublets can be considered.
IKF-575/RENAISSANCE	Randomized Phase III Clinical Trial	In patients with untreated limited-metastatic G/GEJ cancer, 5-FU/leucovorin/oxaliplatin/docetaxel ± targeted therapy followed by radical resection does not improve OS and has increased early mortality compared with standard chemotherapy/targeted therapy.
CABINET	Randomized Phase III Clinical Trial	Cabozantinib improved PFS in patients with previously treated extra-pancreatic/pancreatic NETs compared with placebo. Meaningful toxicity with treatment discontinuation and a lack of an OS benefit are concerning.
KEYNOTE-811	Randomized Phase III Clinical Trial	In patients with HER-2 positive unresectable or metastatic G/GEJ cancer, the addition of pembrolizumab to trastuzumab + chemotherapy improves both PFS and OS.
CHECKMATE-8HW	Randomized Phase III Clinical Trial	First-line nivolumab + ipilimumab showed superior PFS compared with standard chemotherapy in patients with MSI-H/dMMR metastatic colorectal cancer.

## Early detection and diagnostic advances

Innovations in molecular diagnostics and imaging technologies have come a long way and are now on the verge of being utilized for earlier detection of GI cancers. For instance, the detection of circulating tumor DNA (ctDNA) via liquid biopsies has shown promise in detecting minimal residual disease after definitive treatment and monitoring response to systemic therapies.^5-7^ Clinical applicability for ctDNA in GI cancers has been most successful in colorectal cancer (CRC) but is being actively investigated in various oncological settings. The DYNAMIC-Pancreas trial is a prospective cohort study presented at ASCO 2024.^8^ The study enrolled patients with pancreatic adenocarcinoma following upfront surgical resection and employed a tumor-specific ctDNA test to detect somatic mutations for active disease monitoring. After resection of the tumor, those with detectable ctDNA (ctDNA+) were given 6 months of adjuvant chemotherapy (AC), whereas those with undetectable ctDNA (ctDNA−) had the option to receive only 3 months of AC. All treatment was per the clinician’s discretion. From March 2019 to November 2023, 102 patients were enrolled in the study. Of those enrolled, 40% had detectable ctDNA after resection, 53% ctDNA was undetectable, and 4% were unevaluable with no detectable tumor mutation. There was no significant association between ctDNA status and known clinicopathologic risk factors. Among the 54 patients without detectable ctDNA, 24 opted to receive a planned 3 months of AC. With a median follow-up period of 36 months, the median recurrence-free survival (RFS) was 13 months in patients with detectable ctDNA compared with 22 months in those with undetectable ctDNA (HR 0.52, *P* =.003). While detection of ctDNA postresection was associated with a shorter RFS, suggesting ctDNA as a valuable biomarker for prognosticating risk of recurrence, prospective randomized controlled trials to test the true sensitivity, specificity, and clinical utility of the assay in this patient population are needed before this approach is ready for routine clinical practice. Knowing the majority of such patients harbor residual disease, care must be taken to not de-escalate treatment since it may be harmful given the current standard of care is a full 6 months of AC treatment. More likely, early identification of patients at higher risk of recurrence through ctDNA analysis may lead to more innovative therapeutic strategies to improve outcomes.

Also at ASCO, data on using a highly sensitive HPV ctDNA biomarker assay to evaluate clinical outcomes in patients with localized anal cancer after treatment with concurrent chemoradiation (cRT) was presented.^9^ The study collected ctDNA from patients prior to treatment, at the end of treatment (week 5), and at 3, 6, 9, and 12 months posttreatment. Detection of HPV ctDNA 3 months after cRT was significantly associated with clinical recurrence (100% vs 8%; *P* =.006) and inferior RFS (5.9 months vs not reached; *P* <.001). However, differences in RFS based on HPV ctDNA status were not observed at week 5 (*P* =.15). Sensitivity and specificity for recurrence at month 3 were 57% and 100%, respectively, with a positive predictive value and negative predictive value of 100% and 89%. If confirmed, HPV ctDNA status at 3 months post-cRT may serve as an early surrogate marker for disease outcomes allowing future clinical trials to use this as an early endpoint or as a potential go/no-go time point for testing additional interventions or changes in surveillance strategies.

The utility of ctDNA as both a biomarker for recurrence and a prognosticative assay in monitoring disease recurrence appear promising. Whether interventions such as therapy escalation, de-escalation or avoidance based on ctDNA affect clinical outcomes are unanswered and the subject of several ongoing and highly anticipated prospective clinical trials such as CIRCULATE-Japan and TRACC.^10,11^

## Perioperative management

### Neoadjuvant immunotherapy in esophageal/gastric cancer

Immune checkpoint inhibitors (ICIs) have garnered many regulatory approvals due to their efficacy and favorable side effect profile. Also presented at ASCO, the EA2174 trial examined the impact adding nivolumab to neoadjuvant cRT on the pCR rate in patients with esophageal and gastric adenocarcinoma/gastric and esophagogastric junction adenocarcinomas (EGA/GEJ),^12^ and 275 participants with localized EGA/GEJ were randomized to receive neoadjuvant carboplatin and paclitaxel with radiation either without (Arm A) or with (Arm B) nivolumab before surgical resection. The primary endpoint was the pCR rate. Neoadjuvant treatment was initiated in all enrolled patients, and surgery was performed in 78.5% (Arm A: 76.1%, Arm B: 81.0%). pCR rates of 21.0% (95% CI, 14.5-28.8) and 24.8% (95% CI, 17.8-32.9) in Arms A and B, respectively, were not statistically different (*P* =.27). Surgical complication rates were similar between the 2 arms (Arm A: 28.7%, Arm B: 25.4%), with grade 3/4 adverse events occurring in both arms. The findings demonstrate that the incorporation of nivolumab with cRT as neoadjuvant therapy does not affect the pCR rate in patients with resectable EGA/GEJ. This was the first analysis in this 2-by-2 factorial design study with the second analysis (the role of adjuvant nivolumab vs nivolumab with ipilimumab) still anxiously awaited.

### Perioperative chemotherapy in esophageal cancer

The 2024 ASCO Plenary Session featured the ESOPEC trial, a phase III multicenter prospective randomized trial comparing neoadjuvant cRT (CROSS) versus perioperative chemotherapy (FLOT) for resectable locally advanced esophageal adenocarcinoma (EAC),^13^ and 438 participants with resectable EAC from 25 sites in Germany were randomized to receive either CROSS (41.4 Gy plus carboplatin/paclitaxel) or FLOT (5-FU/leucovorin/oxaliplatin/docetaxel), with both regimens followed by surgery. Neoadjuvant treatment was initiated in 403 patients, and surgery performed in 371 with R0 resections achieved in 351 patients. The primary endpoint was OS, with medians of 66 and 37 months for the FLOT and CROSS arms, respectively (HR 0.72, *P* =.023), and 3-year OS rates were 57.4% for FLOT and 50.7% for CROSS (HR 0.70, *P* =.012). The pCR rate was also higher in the FLOT arm (19.3%) compared with the CROSS arm (13.5%). While the study provided a solid case for perioperative FLOT, it is important to note adjuvant nivolumab for residual disease was not employed, a strategy which has shown disease-free survival benefits in CheckMate-577.^14^ This may impact applicability given the contemporary treatment paradigm of esophageal cancer has been increasingly integrating ICI.

## Management of metastatic disease

### Targeted therapy with surgery in esophageal cancer

A multimodal therapy approach for metastatic EGA/GEJ cancer has emerged as a strategy to improve clinical outcomes. While some regiments have been shown to improve OS and PFS,^15,16^ it warrants caution to avoid detrimental side effects from a combination of therapies. The IKF-575/RENAISSANCE trial presented at ASCO 2024 explored the role of surgical intervention in limited-metastatic EGA/GEJ cancer following systemic induction therapy.^17^ In the study, previously untreated patients with limited metastatic disease received 4 cycles of FLOT along with trastuzumab or nivolumab if their tumors were either HER2 or PD-L1 positive, respectively. Patients without disease progression were then randomized to either radical resection of the primary and metastatic lesions prior to additional FLOT therapy (Arm A) or continuation of FLOT therapy alone (Arm B) in lieu of surgical resection(s). A total of 141 patients were randomized, and 139 included in the intention-to-treat analysis (67 in Arm A and 72 in Arm B), and 91% in Arm A underwent surgery with an 82% R0-resection rate. However, higher early mortality in Arm A, led to a crossing of survival curves with 25% and 75% quantiles of OS of 10 versus 14 months and 65 versus 41 months for Arms A and B, respectively. Because of the increased early mortality with surgery, the primary endpoint of OS was not met. Subgroup analyses found that the only group that benefited from the surgery was patients with only retroperitoneal lymph node (RPLN) metastases (median OS of 30 vs 17 months; 5-year OS of 38% vs 19%). While patients whose tumors did not respond to chemotherapy or had peritoneal disease had a statistically significant detriment in survival from the radical resection. This study suggests that the use of multimodal therapy should be selectively considered in the appropriate setting. Resection of the primary in patients with metastatic EGA/GEJ cancer needs further study and may be ill-advised in patients other than those with isolated RPLN metastases.

### Thermal ablation for colorectal liver metastases

Surgical resection colorectal cancer liver metastases (CRLM) has been a mainstay of treatment for decades with clear survival advantages for a subset of patients. However, only a proportion of patients with CRLM are surgical candidates, supporting the assessment of less invasive therapy options as alternative treatment modalities.^18,19^ One promising approach is thermal ablation, which has been shown to be effective in several studies.^20-22^ The COLLISION trial, a multicenter noninferiority trial presented at ASCO 2024 compared thermal ablation to surgical resection for up to 10 potentially resectable small-sized (≤3 cm) CRLM,^23^ and 299 participants were stratified into different subgroups based on disease burden and randomized to either surgery (preferably laparoscopic ± robot over open surgery) or thermal ablation. At a median follow-up of 28.8 months, there was no statistically significant difference in OS between the groups (HR 1.042; 95% CI, 0.689-1.576; *P* =.846), with a 90% probability of noninferiority based on the data. Procedure-related mortality was 2.1% for resection and 0% for thermal ablation, favoring the thermal ablation approach. Moreover, thermal ablation demonstrated fewer adverse events (*P* <.001), shorter median hospital stays (1 day vs 4 days; *P* <.001), and improved local control (HR 0.184; 95% CI, 0.040-0.838; *P* =.029). However, no significant differences were found in local (HR 0.833; 95% CI, 0.473-1.469; *P* =.528) and distant PFS (HR 0.982; 95% CI, 0.739-1.303; *P* =.898). These findings suggest that patients with CRLM have a nonsurgical option, but not that thermal ablation is an option for those that are not surgical candidates. Ongoing studies with more robust statistical analysis beyond noninferiority will assess how thermal and other ablation technologies (eg, SBRT) could be further integrated into clinical practice and exported to a broader group of patients including other cancer types.

### Tumor debulking in multiorgan metastatic CRC

Building on successes in ablative management of oligometastatic CRC,^24,25^ the ORCHESTRA trial also presented at ASCO 2024 was designed to address whether aggressive tumor debulking could offer improvements in outcomes.^26^ Based on multidisciplinary discussions, this phase III study enrolled 454 patients with metastatic CRC for whom at least 80% tumor debulking was deemed feasible. After initial systemic therapy with capecitabine or 5-fluorouracil/leucovorin and oxaliplatin plus bevacizumab, 382 patients with clinical benefit were randomized to either continue with systemic therapy alone (standard arm, *N* = 192) or undergo aggressive tumor debulking followed by systemic therapy (experimental arm, *N* = 190). The median follow-up was 32.3 months. Median OS values of 27.5 and 30.0 months in the standard arm and experimental arms, respectively, were not statistically difference (adjusted HR 0.88; 95% CI, 0.70-1.10; *P* =.225). Median PFS was also similar, at 10.4 months in the standard arm and 10.5 months in the experimental arm (adjusted HR 0.83, 95% CI, 0.67-1.02, *P* =.076). The study suggests that palliative surgical debulking offers limited clinical benefit for patients with metastatic CRC.

### Targeted therapy in neuroendocrine tumors

Given targeting angiogenesis in neuroendocrine tumors (NETs),^27,28^ has had limited success, the CABINET trial assessed the multikinase inhibitor cabozantinib in patients with advanced NETs, with updated results presented at ESMO 2024,^29^ and 203 patients who had tumor progression within 12 months before registration and at least 1 prior systemic therapy were randomized 2:1 in 2 separate cohorts based on pancreatic/extrapancreatic NETs to either cabozantinib or placebo. The trial terminated early due to interim analysis showing statistically significant PFS benefits with cabozantinib in both cohorts. For the extrapancreatic NET cohort, the median PFS was 8.5 months with cabozantinib versus 4.0 months with placebo (HR 0.38; 95% CI, 0.25-0.58). For the pancreatic NET cohort, the median PFS was 13.8 months versus 4.5 months (HR 0.23; 95% CI, 0.12-0.42). The objective response rate (ORR) was also higher in the cabozantinib arm (5% in the extrapancreatic cohort, 19% in the pancreatic cohort, and 0% in the placebo cohort). Treatment-related adverse events, including Grade 3 or higher events, were more frequently observed in the cabozantinib arm (62% vs 23%). The lack of an overall survival benefit and the marked cabozantinib toxicities that led to frequent treatment discontinuation leave the value of this therapy in question.

## Emerging roles of immunotherapy

### Immunotherapy in metastatic CRC

Accumulating evidence has ratified that immunotherapy efficacy is higher in tumors with mismatch-repair-deficiency (dMMR) and microsatellite instability (MSI-H).^30,31^ The strong association of these tumors with Lynch Syndrome has led to their more frequent detection in patients with colorectal and endometrial cancers with promising results in dMMR/MSI-H CRC.^32^ For example, the combination of nivolumab plus ipilimumab has demonstrated clinically durable responses in MSI-H or dMMR metastatic CRC.^33,34^ Building on this compelling evidence, CheckMate 8HW, a randomized, phase III open-label trial, was designed to compare the efficacy of nivolumab alone and nivolumab plus ipilimumab against standard first-line chemotherapy. An update on additional subgroup efficacy and expanded safety analyses from the trial was presented at ESMO 2024.^35^ A total of 303 patients were randomized, with 202 receiving nivolumab plus ipilimumab and 101 receiving chemotherapies. MSI-H/dMMR status was centrally confirmed in 171 patients in the nivolumab plus ipilimumab arm and 84 in the chemotherapy arm.

With a median follow-up of 31.5 months, PFS significantly favored nivolumab plus ipilimumab across all key subgroups of patients with centrally confirmed MSI-H/dMMR. Median PFS was not reached in the nivolumab plus ipilimumab arm compared with 5.8 months in the chemotherapy arm (HR 0.21, 95% CI, 0.13-0.35, *P* <.0001). Consistent benefits were also observed in specific subgroups including patients with liver metastases (HR 0.11; 95% CI, 0.05-0.25), peritoneal metastases (HR 0.19; 95% CI, 0.10-0.34), and those with KRAS/NRAS mutations (HR 0.24; 95% CI, 0.09-0.63). In contrast, patients with MSS/pMMR did not derive benefit, with median PFS favoring chemotherapy in this group. The trial is ongoing, with an anticipated update expected to compare nivolumab alone to nivolumab plus ipilimumab. Given the encouraging results observed in the nivolumab plus ipilimumab arm and its well-tolerated side effect profile across the cohort, it is foreseeable that the treatment approach for MSI-H/dMMR metastatic CRC may soon include doublet ICIs, pending the final result of the study likely available in 2025.

### Immunotherapy combined with HER-2 directed therapy in metastatic esophageal cancer

Given the significant clinical responses observed with anti-PD-L1 therapies in CPS > 1 tumors and HER-2 therapies in HER-2 positive disease when individually added to standard chemotherapy regimens in advanced or metastatic G/GEJ adenocarcinoma,^36,37^ their use in combination has been investigated for HER2-positive advanced or metastatic G/GEJ adenocarcinoma in KEYNOTE-811. The updated result on OS benefit was reported in ESMO 2024.^38^ In this randomized, double-blind trial, a total of 698 patients with histologically confirmed HER-2 positive metastatic G/GEJ adenocarcinoma were randomized in 1:1 to trastuzumab + standard of care chemotherapy with or without pembrolizumab. With a median follow-up of 50.2 months, the investigators reported a significant improvement in OS with the addition of pembrolizumab (median 20.0 vs 16.8 months; HR 0.80; 95% CI, 0.67-0.94; *P* =.0040), reinforcing a PFS benefit of 10.0 versus 8.1 months; HR 0.73; 95% CI, 0.61-0.87. The ORR was also higher with the addition of pembrolizumab (72.6% vs 60.1%), and grade ≥ 3 drug-related adverse events occurred in 59% versus 51%. While combined therapy has been widely adopted as first-line in HER2-positive metastatic G/GEJ adenocarcinoma, these updated findings further solidify and position pembrolizumab in combination with trastuzumab and chemotherapy as a standard of care for this patient population.

## Systemic treatment switch strategies

### Maintenance therapy in advanced gastric cancer

In the absence or low expression of targetable receptors such as HER-2, PD-L1, or claudin 18.2, platinum and fluoropyrimidine doublets have remained the standard first-line therapy in advanced gastric cancer for almost 2 decades ago.^39-41^ Maintenance therapy has emerged as a strategy to prolong the benefit of the initial treatment and mitigate treatment-limiting toxicities. This is relevant considering the limited survival benefits of second-line therapy.^42^ The ARMANI study, a phase III clinical trial presented at ESMO investigated the efficacy of switch maintenance therapy from initial oxaliplatin-based chemotherapy to either ramucirumab plus paclitaxel (arm A) or continued CAPOX/FOLFOX for an additional 3 months, followed by fluoropyrimidine monotherapy maintenance (arm B), in patients with HER2-negative advanced gastric/GEJ cancer without disease progression after 3 initial months of treatment.^43^ With 280 patients randomized, at a median follow-up of 43.7 months, median PFS was 6.6 months with switch therapy (arm A) versus 3.5 months with standard fluoropyrimidine maintenance (arm B) (HR = 0.63, 95% CI, 0.49-0.81; *P* <.001). Median OS was 12.6 months in arm A versus 10.4 months in arm B (HR = 0.75, 95% CI, 0.58-0.97; *P* =.030). However, the frequency of grade 3 or higher adverse events was higher in arm A (40.4%) compared with arm B (20.7%), with notable differences in neutropenia (25.5% vs 9.6%), hypertension (6.4% vs 0%), and venous thromboembolism (2.1% vs 0%). While the findings from the ARMANI trial suggest a second-line therapy option such as ramucirumab and paclitaxel as maintenance therapy before disease progression in the first line could lengthen disease control and improve survival, the notable side effects are concerning and require close monitoring and mindful patient selection.

## Conclusion

This collection of trial results from the 2024 ASCO and ESMO Annual Meetings demonstrate continued progress in the therapy of GI cancers across multiple fronts from molecular diagnostics using ctDNA as a prognostic marker across multiple GI tumor types to localized treatments such as thermal ablation and perioperative chemotherapy. It is encouraging to note the expanding benefits and utilities of immunotherapy and targeted therapies in GI cancers. With the collective goal of improving patient care and outcomes, the ongoing efforts of the cancer research and patient community provide hope for continued progress toward improved patient outcomes and new standards of care.

## Data Availability

There are no new data associated with this article
